# Development of Small Molecules Targeting α-Synuclein Aggregation: A Promising Strategy to Treat Parkinson’s Disease

**DOI:** 10.3390/pharmaceutics15030839

**Published:** 2023-03-03

**Authors:** Samuel Peña-Díaz, Javier García-Pardo, Salvador Ventura

**Affiliations:** 1 Institut de Biotecnologia i Biomedicina, Universitat Autònoma de Barcelona, 08193 Bellaterra, Spain; 2 Departament de Bioquímica i Biologia Molecular, Universitat Autònoma de Barcelona, 08193 Bellaterra, Spain

**Keywords:** Parkinson’s disease, aggregation, α-Synuclein, amyloid, inhibitor

## Abstract

Parkinson’s disease, the second most common neurodegenerative disorder worldwide, is characterized by the accumulation of protein deposits in the dopaminergic neurons. These deposits are primarily composed of aggregated forms of α-Synuclein (α-Syn). Despite the extensive research on this disease, only symptomatic treatments are currently available. However, in recent years, several compounds, mainly of an aromatic character, targeting α-Syn self-assembly and amyloid formation have been identified. These compounds, discovered by different approaches, are chemically diverse and exhibit a plethora of mechanisms of action. This work aims to provide a historical overview of the physiopathology and molecular aspects associated with Parkinson’s disease and the current trends in small compound development to target α-Syn aggregation. Although these molecules are still under development, they constitute an important step toward discovering effective anti-aggregational therapies for Parkinson’s disease.

## 1. Introduction

An increasing number of human disorders are being linked to the formation of abnormal, stable, and usually toxic protein aggregates. Protein aggregation is a heterogenous process that conduces the formation of multiple structures, among which one of them has attracted most of the attention. Amyloid aggregates are characterised by a fibrillar morphology that can be observed using Transmission Electron Microscopy (TEM) or Atomic Force Microscopy (AFM) [1,2]. Interestingly, although different unrelated sequences can form amyloid fibrils with diverse general conformations, they all share a highly ordered architecture. These aggregates are composed of a cross β-sheet structure sustained by parallel or anti-parallel β-sheets that run perpendicularly to the fibril axis [3]. Amyloid structures have been traditionally considered to be pathological. Nevertheless, several amyloids have also been described to play an important functional role in living organisms [4,5,6,7,8,9], such as human premelanosome protein (PMEL17) [10], RNA-binding proteins [9,11]**,** or some human protein hormones, including glucagon- and corticotropin-releasing hormones [12]. Thus, amyloid fibrils are two sides of the same coin. These proteins might perform essential roles such as structural support, storage, signaling, or adaptation functions or being responsible for severe pathologies, including spongiform encephalopathies, Alzheimer’s disease (AD), Parkinson’s disease (PD), amyotrophic lateral sclerosis, Huntington’s disease, or type II diabetes, among others [13].

PD is considered to be the world’s second most prevalent neurodegenerative disorder, after AD [14,15]. This disease exhibits a global prevalence of 0.3% and affects 2–3% of the population above 65 years of age [16]. PD is a complex pathology initially associated with motor deficiencies, as a result of an acute neuronal loss in substantia nigra pars compacta (SNc), with a significant dopaminergic (DA) impairment [17]. Nevertheless, PD has also been related to non-motor symptoms (NMS) that increase the pathology’s severity [18,19]. The cause of PD is still unknown; however, its origin seems to result from a combination of environmental and genetic factors [20], with the presence of intracellular proteinaceous clusters, mainly composed of α-Synuclein (α-Syn), as the major neuropathological hallmark [21]. PD therapies are currently focused on alleviating the motor symptomatology [22,23]. Remarkably, PD is not the only disease associated with α-Syn amyloid formation. In disorders such as Multiple System Atrophy (MSA) or Dementia with Lewy Bodies (DLB), abnormal α-Syn accumulations in the brain cells have been described [24,25,26]. Together, these pathologies constitute a heterogeneous group of disorders, known as synucleinopathies, which share common neuropathological features, but differ in the cellular and anatomical location of α-Syn aggregates [26,27,28] and the symptomatic development. Nowadays, there is no effective treatment for these diseases.

In the present review, we provide a historical overview of the physiopathology and molecular aspects associated with PD development, focusing on α-Syn dysfunction and aggregation. Furthermore, we explain the current trends in small compound development to target α-Syn aggregation and their implications in drug discovery. Finally, potential future directions for PD pharmacologically are also discussed.

## 2. Historical Overview

**Figure 1:**
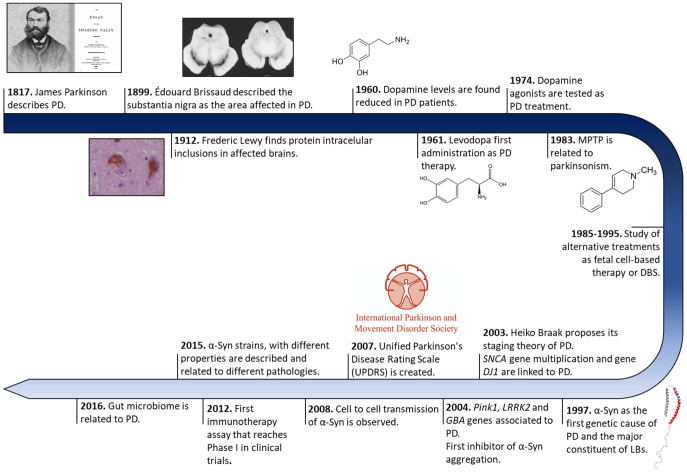
Historical overview of Parkinson’s disease research. Schematic representation of some of the most relevant findings in PD research from its discovery to treatment development.

The history of PD began two centuries ago in England (Figure 1). In 1817, James Parkinson published a book entitled ‘An Essay on the shaking palsy’. In this treatise, Parkinson methodically described the development of a disorder in six patients with a motor disability, observing resting tremors, paralysis, and an unnatural posture [29]. Sixty years later, in 1872, the neurologist Jean-Martin Charcot, whose work helped to differentiate between bradykinesia, stiffness, and weakness in PD [30], named the disorder Parkinson’s disease in honor of James Parkinson. Two decades later, in 1893, Blocq and Marinescu analyzed a patient exhibiting resting tremors due to a granuloma that affects the SNc [31]. Finally, in 1899, Brissaud suggested that SNc is the most affected region in PD-suffering patients [32]. However, it was not until 1919 that the first pathological evidence was obtained by Trétiakoff who provided a description of significant neuromelanin loss in SNc neurons and the presence of Lewy bodies (LBs) [33], cytoplasmatic structures previously reported by Frederic Lewy in PD-affected brains [34]. Altogether, DA cell loss and LBs presence in SNc constituted the first anatomical proof of PD and allowed its post-mortem diagnosis [35].

Later, in 1957, Arvid Carlsson made a discovery that would play a key role in PD treatments. Carlsson described, in animals, the role of dopamine in motor activity under the control of the basal glial, whose deficiency could be reverted by L-3,4-dihydroxyphenylalanine (*L*-DOPA) administration [36]. Three years later, Ehringer and Hornykiewicz described a dopamine deficiency in the striatum and SNc of PD-affected brains [37]. During the following years, *L*-DOPA intravenous or oral administrations were extensively investigated [22], and many authors reported significant improvements in motor symptoms (MS) [23], thus it became the default therapy for PD.

Eighty years after the discovery of LBs, a protein called α-Syn was found to be the major component of these cytoplasmatic structures [21]. These findings correlated with previous evidence of genetic mutations in the *SNCA* gene (that encodes for α-Syn), the first gene described as a genetic cause of PD [38]. Since then, other numerous genetic alterations in *SNCA* (single-point mutations, duplications, and/or triplication) [39,40,41,42,43,44,45] and other genes (*GBA*, *PINK1*, *LRRK2*, *Parkin,* or *DJ1*, among others) [46,47,48,49] gradually appeared as risk factors of PD early onset and progression, opening up possibilities for the development of genetic models that recapitulate the molecular origin the disease better than the 1-methyl-4-phenyl-1,2,3,6-tetrahydropyridine (MPTP) or 6-hydroxydopamine (6-OHDA) neurotoxin ones do [50]. These new cellular and animal models have allowed the study of α-Syn transmission, therapeutic approaches, and biomarkers, especially for the initial stage of PD (prodromal stage) [51,52,53,54,55]. Currently, the search for effective treatments for PD has been mainly focused on α-Syn aggregation [56], but genetic therapies and microbiota alterations linked to PD are receiving increasing attention [57,58].

## 3. Symptomatology

PD has been traditionally considered as a neuronal disorder with symptomatology that is limited to unilateral and asymmetric motor deficits such as rigidity, tremors, bradykinesia, and postural instability. Clinical diagnosis has been based on bradykinesia and other cardinal motor deficiencies [59,60]. The onset of these symptoms varies between <40 and >80 years of age [16]. A young onset of PD is considered to occur at an age < 45 and is usually correlated to genetic factors [61,62]. However, PD involves additional clinical features with NMS, such as sleep disorders, cognitive impairment, depression, anxiety, pain, or dementia [63,64,65,66], which significantly aggravate the patient’s quality of life [67]. The development of PD impacts numerous neurotransmission pathways, which could explain the appearance of these NMS; for example, depression could be related to the deterioration of cholinergic, noradrenergic, and serotonergic systems, while DA and noradrenergic decay would induce anxiety [68,69,70,71,72].

**Figure 2:**
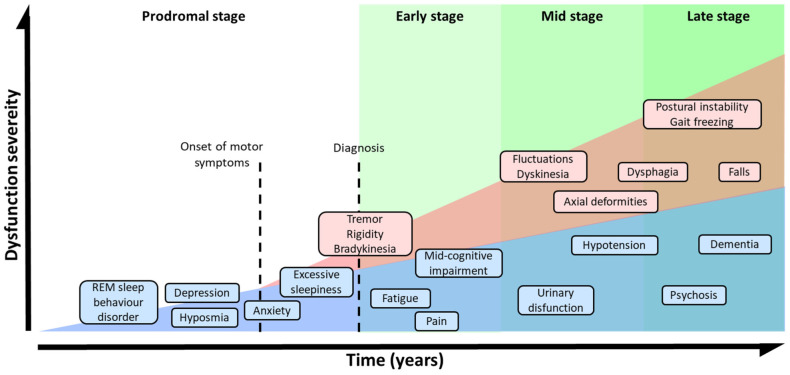
Symptomatic progression of PD. Schematic overview of both, MS and NMS symptoms progression and variability. PD diagnosis is based in MS, but NMS usually appears years before MS could be appreciated during the prodromal stage. The severity of the symptoms results from a combination of NMS, MS, and *L*-DOPA-derived complications.

The absence of MS and the presence of symptoms such as olfactory dysfunction, REM sleep behavior disorder, constipation, and depression are indicators of the prodromal stage of PD [63]. The development of these NMS during the prodromal phase precedes MS by several years [63,73,74] (Figure 2). In the early stages of the disorder, patients develop bradykinesia, tremors, and rigidity, and up to 21% of them also experiment pain, depression, or anxiety [75]. Initially, the disease can be treated with symptomatic therapies, but as it progresses, the treatment becomes more complicated [61,76]. In the latest stages, patients exhibit severe NMS such as dementia (83%), hallucinosis (74%), orthostatic hypotension (48%), urinary incontinence (71%), or constipation (40%), leading to a pronounced disability [76]. In addition, these phases are characterized by a progressive physical incapacity and strong resistance to the treatments, inducing freezing gait, postural instability, falls, and choking [77].

## 4. Risk Factors and Genetics of Parkinson’s Disease

Neurodegenerative disorders constitute a diverse group of pathologies, with many challenges that need to be faced. Identifying the leading cause of the disease onset and progression is one of the major pending questions, and PD is a paradigmatic example. Although the intrinsic cause of PD onset and development remains uncertain, numerous factors (from environmental to genetic ones) have been reported to play a role. In the case of environmental factors, meta-analyses of case–control sets have described environmental factors as both increasing (pesticide exposure, prior head injury, rural living, β-blocker use, agricultural activities, and well-water consumption) and decreasing (tobacco, coffee, non-steroidal anti-inflammatory drug, and calcium channel blocker consumption and alcohol abuse) the risk elements for developing PD [78]. 

Even though aging is still considered to be the major risk factor, genetic studies have revealed that defects in numerous genes also play a significant role in the onset and evolution of PD. The genetic alterations commonly associated with familial cases of the pathology have been summarized in Table 1. The first gene linked to PD was *SNCA*, which encodes for α-Syn and includes numerous single-point mutations. The first described *SNCA* mutation, A53T [40], was initially detected in patients that presented an accelerated course of the pathology, manifesting cognitive impairment after 5–7 years of onset and an average onset of 46 years [79,80]. One year later, researchers observed another single-point mutation: A30P [39], whose clinical profile, interestingly, revealed a more benign course of the disorder and a later onset. In the case of the E46K mutation [41], patients develop severe symptoms between the ages of 50 and 65, including dementia and autonomic failure [41,81]. Another genetic mutation, H50Q [43], was first described in patients that reported motor symptoms at the age of 60 and fast development of the pathology [43]. In the last decade, two other genetic mutations, G51D and A53E [44,82], have been reported to cause dementia and autonomic dysfunction and early motor symptom progression, respectively [44,83,84,85,86,87]. Furthermore, other single-point mutations, such as E83Q and A30G, which enhance α-Syn aggregation, have recently been described in PD patients [88,89]. *SNCA* duplications and triplications have also been observed in inherited PD. In this case, the disorder’s severity and progression are related to the number of gene copies, resulting in more severe symptomatology and early onset in patients affected by triplications of *SNCA* than those affected by duplications [42].

Other autosomal dominant mutations described in PD include genes with diverse functions. For example, missense mutations in the *LRRK2* gene, which encodes for a large kinase with various functions such as vesicle trafficking or GTPase activity [90,91], have been identified in PD patients worldwide [49,92,93]. The mutant LRRK2 induces apoptotic neuroblastoma and cortical neurons death probably by altering the autophagy process [94,95]. Nevertheless, the most common genetic alterations, with more than 300 different mutations described, are observed in the *GBA* gene [96,97,98], which encodes for a lysosomal enzyme called glucocerebrosidase that degrades glucosylceramide into glucose and ceramide [99,100,101]. The role of *GBA* mutations in PD onset and progression is still under debate. Theories such as impaired lysosomal function or endoplasmic reticulum-associated stress have been related to *GBA* mutations in PD, but the accumulation of α-Syn is considered to be the most plausible hypothesis [102,103]. *GBA* carrier patients exhibit an early onset of PD with an acute motor deficit, but these mutations notably increase the severity of NMS, enhancing cognition impairment, depression, and anxiety, among other symptoms [97]. 

Although autosomal dominant mutations are the most common genetic factors of PD, autosomal recessive alterations have also been related to this pathology. The *parkin* gene, which encodes for an E3 protein–ubiquitin ligase, is one of these recessive mutants, including an altered number of gene copies and missense and nonsense mutations [104]. Parkin ligase regulates the degradation of misfolded proteins through the ubiquitin–proteasome system [105] and interacts with LRRK2 [106]. The role of these mutations in the development of the disease seems to relate to the accumulation of damaged mitochondria [107]. *DJ1* and *PINK1* mutations present similar features to those of parkin, as they participate in common biochemical pathways [45,107,108,109,110], differing in the presence of diffuse or complete LBs and Lewy neurites (LNs), respectively. On the one hand, mutations of *DJ1*, a modulator of gene expression under cellular stress [111,112,113], induce DJ1 migration to the outer mitochondrial membrane, increasing the sensitivity to stress [111,112]. On the other hand, *PINK1* gene alterations include missense, nonsense, and splice mutations or small deletions or insertions [114,115] that induce a mitochondrial deficit and alter the mitophagy pathways [47].

## 5. Molecular Mechanism Implicated in PD Development

**Figure 3:**
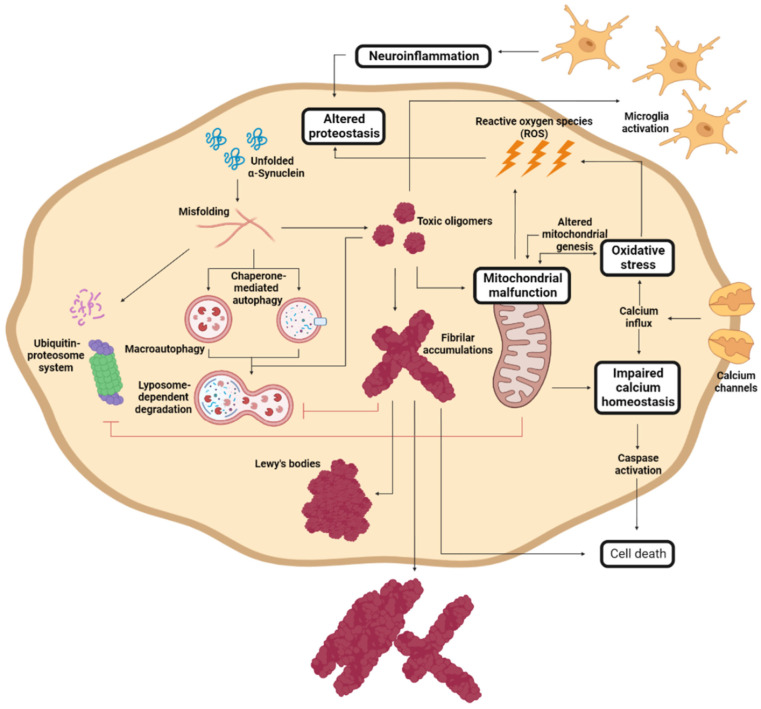
Molecular mechanism of Parkinson’s disease cell damage. Schematic representation of the interconnected molecular processes that induce cell death and PD progression. Adapted from [61].

During PD progression, cells suffer numerous interconnected and retrograde alterations that culminate in the degeneration of specific brain regions (Figure 3). α-Syn seems to be a critical element around which most molecular and genetic factors converge [116,117,118,119]. In pathogenic conditions, α-Syn forms neurotoxic oligomeric structures that progressively assemble into insoluble fibrils and accumulate in LBs [21,116,117]. Recent studies have demonstrated that these aggregates could be transmitted to neighboring cells, seeding the aggregation of the protein in healthy neurons, and spreading the disease to different brain regions [120,121,122,123].

α-Syn proteostasis depends on the ubiquitin–proteasome and the lysosomal autophagic systems [119,124], whose inhibition leads to the accumulation of α-Syn [118,125]. Accordingly, mutations in the *LRRK2*, *GBA*, and/or *VPS35* genes translate into a pronounced number of LBs and LNs [101,126,127,128]. Conversely, the pharmacological stimulation of autophagy systems significantly decreased the aggregated α-Syn in animal models [129,130]. Oligomeric α-Syn and its accumulation alter the function of the ubiquitin–proteasome system, inhibiting macroautophagic processes and chaperone-mediated autophagy [131].

The aggregation of α-Syn also induces mitochondrial dysfunction, which stimulates the formation of amyloid fibrils [94]. α-Syn accumulation decreases the levels of peroxisome proliferator-activated receptor-γ co-activator 1α, a mitochondrial regulator of the transcription [132,133], which reduces the levels and toxicity of α-Syn oligomers when it is activated [134]. This relates to the relevance of LRRK2 (mutations leading to mitochondrial impairment), Parkin, and PINK1 (responsible for the degradation of harmed mitochondria) in PD development [94,110]. As a result of mitochondrial dysfunction, there is a significant accumulation of metabolites that produce high oxidative stress levels [135], to which unmyelinated DA neurons are susceptible [136,137]. Mutations of DJ1 significantly reduce the cellular response to stress [108,111,112]. Mitochondrial deficiency also impacts the energy levels of the neuron, inducing impaired calcium homeostasis and rapid axonal degeneration [138,139]. 

Another molecular process that plays an important role in PD and that is closely related to α-Syn aggregation and spreading involves immune system activation [140]. α-Syn aggregation stimulates an adaptative and innate immune response to toxic amyloids, but, at the same time, the generated neuroinflammation retroactively enhances protein aggregation [141,142,143], aggravating neuroinflammation. Therefore, modulating the immune response to toxic α-Syn species has been extensively studied as a therapeutic alternative.

## 6. Alpha-Synuclein

**Figure 4:**
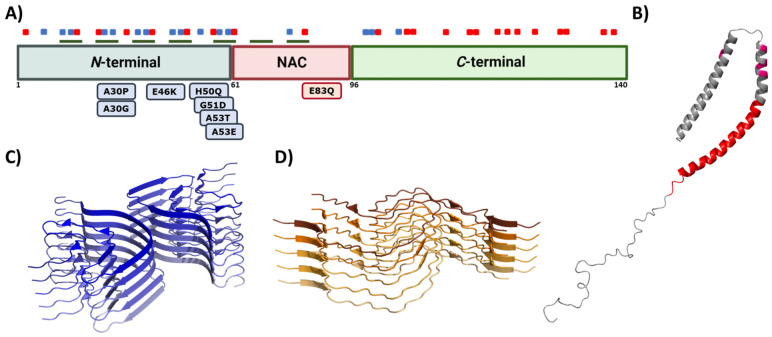
Alpha synuclein architecture. (**A**) Schematic representation of α-Syn primary sequence indicating the location of positively (blue) and negatively (red) charged amino acid, and KTKEGV repeats (green). Sequential domains and single-point mutations related to familial cases of PD are also indicated below the linear representation. (**B**–**D**) Structure of monomeric (**B**) and aggregated α-Syn forming different conformations or strains (**C**,**D**). In (**B**–**D**), the PDB files used are: 1XQ8, 6CU7, and 6CU8, respectively.

Due to the connection between α-Syn and PD onset and progression, this protein has become the preferred target in the search for a disease-modifying treatment [56]. α-Syn is a 140 amino acid protein encoded in *SNCA* gene and is mainly expressed in the brain’s synaptic termination of DA neurons. In normal conditions, α-Syn can be found as a soluble, monomeric, and disordered protein or bound to lipidic membranes, with an alpha-helical conformation (Figure 4B) [144]. Some studies have also revealed the possibility that α-Syn forms a tetrameric and helical structure in the cytoplasm [145]. Although its function remains unclear, it has been related to vesicle trafficking at the synapsis [146], participating in the release and recycling processes. This activity might be mediated by its interaction with VAMP2, a synaptobrevin involved in the fusion and binding of synaptic vesicles [147]. This interaction stabilizes SNARE complexes, which intervene in vesicle fusion and neurotransmitter release [148,149]. Nevertheless, α-Syn might also play alternative functions. For example, some studies suggest that α-Syn stabilizes the mRNA in P-bodies by binding proteins found at these membrane-less organelles [150], while others suggest that α-Syn may modulate DNA repair [151].

This multivalent activity is connected to its particular sequence, which could be dissected into three different regions (Figure 4A) [152]. The *N*-terminal domain is a highly conserved protein region that concentrates the most imperfect KTKEGV repeats. These repeats confer an amphipathic character responsible for the conformational change to an α-helical configuration and the protein–lipid interaction that dictates the binding to the membranes [144,153]. Significantly, this protein–lipid interaction has been described as a risk factor when the concentration of α-Syn increases, as it facilitates local nucleation for amyloid formation [154,155,156,157]. Recent studies have also proved that the *N*-terminal region contains two sequences (residues 36–42 and residues 45–57) with an essential role in homomeric α-Syn interactions, establishing contacts between monomeric α-Syn proteins that precede cross-β formation [158,159,160]. In addition, most of the missense mutations related to the early onset of PD, MSA, or DLB are also found in this region [38,39,40,41,43,44]. The central region is also known as the Non-Amyloid Component (NAC), as it is an important component of amyloid plaques in AD [161]. This is a hydrophobic segment often protected by the transient interactions that occur because of the disordered nature of the protein, but it drives the aggregation of α-Syn in pathogenic conditions [25,162,163,164]. In contrast, the *C*-terminal domain presents a large amount of acidic amino acids that provide a highly negative charge density. This net charge seems to chaperone α-Syn aggregation by electrostatic repulsions [165]. Accordingly, *C*-terminal truncations of α-Syn increase the aggregation propensity and toxicity and are important components of LBs, which suggests that this process could play a relevant role in pathogenesis [24].

**Figure 5:**
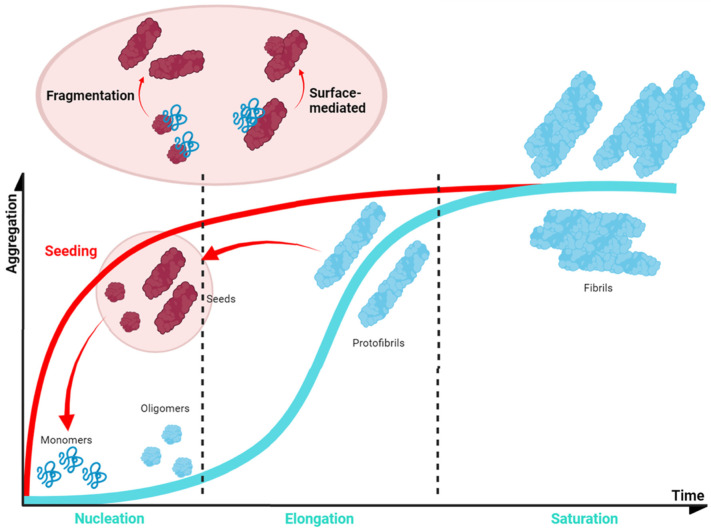
Schematic α-Syn aggregation profile. The aggregation kinetics of most of the proteins can be dissected into three main phases (blue). This also applies to α-Syn, for which the first step (nucleation or lag phase) is characterised by the formation of small nucleus that would guide the process; these nuclei incorporate monomeric protein prompting an exponential growth of the aggregate (elongation or exponential phase); finally, the system enters in an equilibrium in which mature fibrils could be observed (saturation or plateau phase). However, the process could be accelerated (red) as fibrils can fragment into smaller aggregates that can be incorporated at the initial stages as nuclei or seeds (seeding).

In normal conditions, α-Syn displays significant solubility, but in pathogenic situations, this protein tends to establish β-sheet interactions that induce the formation of insoluble amyloid-toxic structures, compromising cellular homeostasis and inducing neuronal death [166], as outlined above. Amyloid aggregation is a complex process that, in vitro, often can be assimilated to a sigmoidal representation with three different phases, reflecting a nucleation–polymerization process [167]. During the lag phase, α-Syn monomers interact, forming toxic and transmissible structures named oligomers and protofibrils [168] that will act as the aggregation nuclei. The second step, or the elongation phase, consists of an exponential increase in the number and size of the fibrils. Finally, the plateau phase is characterized by the presence of mature and long amyloid fibrils (Figure 5). However, this is a very simplified description of the aggregation process, which in addition comprises alternative events caused by fibril fragmentation (secondary nucleation) or seeding processes, which contribute to accelerate or abrogate the nucleation phase (Figure 5) [169].

Genetic and environmental factors further modulate the aggregation process of α-Syn. Missense mutations of α-Syn (A30P, E46K, H50Q, G51D, A53E, and A53T), which are located at the membrane-binding region (Figure 4A) and impact the protein aggregation propensity, inducing either its oligomerization (A30P, H50Q, and A53T) or fibril formation (H50Q, A53T, E46K, and E83Q), and, thus, the formation of α-Syn toxic species [170]. In contrast, G51D and A53E variants slow down α-Syn aggregation compared to that of wild-type (WT) α-Syn, but they alter its interaction with the membranes [82,171]. Despite the exact role of these mutants in PD is still unknown, patients suffering G51D and A53E mutations exhibit a large amount of α-Syn pathological inclusions (in the case of G51D, also in oligodendrocytes) [44] and an earlier onset of PD. Regarding this evidence, it is suggested that these missense mutations could prolong the lifetime or stimulate the generation of toxic α-Syn structures such as oligomers [82]. Moreover, recent studies have shown that environmental factors, such as ionic strength or pH, affect the intermolecular interactions of α-Syn and contribute to the heterogeneity of the aggregation process. The analysis of the effect of pH variations on α-Syn aggregation suggested that acidic conditions (ranging from pH = 5 to pH = 3) induce the formation of partially folded species containing the β-sheet conformation, while retaining the monomeric ones [172]. This partial folding, as reported in terms of Thioflavin-T (Th-T) kinetics, prompts a significant decrease in the lag phase, while increasing the elongation rate, and, thus, stimulating α-Syn aggregation probably by impacting the protonation state of the *C*-terminal domain [172]. Regarding ionic strength, several studies have been performed to elucidate the role of salt in α-Syn aggregation. On the one hand, when α-Syn is incubated in the presence of preformed fibril seeds (PFFs) or lipid vesicles, the seeded polymerization of the protein notably decreased with an increasing salt concentration [173]. In contrast, when monomeric α-Syn is aggregated in the absence of these seeds, the salt significantly promotes the aggregation of WT and familial variants of α-Syn and impacts the fibrillar structure [174,175,176]. Although both results may seem to be contradictory, they are, in fact, complementary. As reported in several studies [117,177,178], the presence of salt during the aggregation process compensates for the electrostatic repulsions exerted by the *C*-terminal region, thus precluding the anti-aggregational effect of this region and accelerating protein aggregation. Nevertheless, the fibrils obtained in the presence of salt presented a higher level of compaction than the ones formed in its absence do, which has been suggested to result from the hiding of the C-terminal acidic domain, which is normally exposed and forms a fuzzy coat. Interestingly, the presence of these disordered regions on the fibril might play a relevant role in seeded polymerization. When this region is exposed at the PFFs surface, it has been reported to promote the amyloid aggregation of α-Syn either in vitro or in cellular models. In these cellular models, the *C*-terminus facilitates PFFs internalization by interacting with cell surface receptors [117,177,178]. Accordingly, and as for human prion proteins [179,180], α-Syn fibrils formed under different solution conditions share a common cross-β fold, but exhibit different conformation, seeding activity, neurotoxicity, and spreading in cells and when they are inoculated in rat brains [177,181,182,183,184,185]. These diverse conformational assemblies are called strains (Figure 4C,D) and could explain the existence of different synucleinopathies with unique clinical features [182,186,187] as their different properties would induce particular lesion profiles and brain region dissemination [188]. Indeed, a recent Cryo-EM comparative study evidenced structural differences between α-Syn fibrils obtained from MSA patients and those obtained from patients with DLB [186], supporting the existence of functionally distinct strains in humans. 

Recent studies have suggested that in addition to aggregation, α-Syn can undergo liquid–liquid phase separation (LLPS) in vitro and in vivo as a previous step to amyloid formation [189,190,191]. LLPS is a recently described aggregation-related phenomenon characterized by the formation of multivalent macromolecular interactions, which induce the formation of an alternative phase with particular physicochemical properties that may be the main responsible for the formation of membrane-less organelles [192,193,194,195,196,197]. The mechanism underlying the transition of LLPS to amyloid is still unclear, with only a few structural studies addressing it [191]. Environmental conditions, such as pH or ionic strength, also condition LLPS and the maturation into aggregates [198]. This might be the intrinsic mechanism that accounts for the formation of different strains. Remarkably, environmental risk factors for PD, such as the Ca^2+^ or Mn^2+^ cations, have been proven to facilitate LLPS and accelerate aggregation [199,200]. Thus, even though the functional role of α-Syn in LLPS remains unclear, understanding the interactions that lead to amyloid transition might permit the stabilization of LLPS and prevent further progress toward aggregation [201]. Moreover, new studies have suggested that α-Syn and other amyloid-like proteins, such as prion and tau, may be synergistically connected via LLPS with different amyloidosis [202,203].

In regard to in vivo studies, the formation of α-Syn fibrillar structures is preceded by the assembly of the monomeric protein into small and diffusible metastable oligomers and protofibrils, which have been suggested to be the main culprits of neuronal degeneration [168,204,205,206,207,208,209]. These aggregated, but diffusible, structures progressively appear in different brain regions [210], consistent with a prion-like transmission mechanism as proposed by the Braak’s theory. This hypothesis suggests the development of PD follows a sequential pattern, beginning in the dorsal motor nucleus of the vagus nerve in the brainstem, and then spreading to other areas of the brain, which would explain the gradual development of different PD symptoms [120,211].

## 7. New Therapies: Modulating α-Synuclein Aggregation

The lack of an effective therapy targeting PD’s molecular basis has led to a continuous search for new treatments. One of these approaches is gene therapy, which has become a relevant strategy for treating numerous diseases. Lentiviral and adeno-associated viral vectors that have been approved for human use [212,213,214] are the most studied in PD, with different targets being identified for possible gene treatments, including disease modifiers and non-modifiers. Glutamic acid decarboxylase (GAD) overexpression through adeno-associated vectors administration was the first gene therapy studied in PD patients. It improved their symptomatic profile, but did not result in neuroprotective activity [215]. Other studied therapies are based on L-amino acid decarboxylase gene administration, alone or combined with tyrosine hydroxylase and GTP cyclohydroxylase 1 [216,217]. The L-amino acid decarboxylase gene plays a crucial role in dopamine metabolism, but genetic therapies targeting this gene only resulted in a Unified Parkinson’s Disease Rating Scale (UPDRS) score improvement [216,217]. Alternative gene therapies are based on the overexpression of growth and/or neuroprotective factors, such as glial cell-line derived neurotrophic factor, neurturin, artemin, persephin, vascular endothelial growth factor, or Nurr1 [218,219,220,221]. It has also been suggested that CRISPR/CAS9 technology could be used to correct genetic mutations associated with PD [222]. Other studied approaches were based on cellular transplantation. During the 1990s, fetal cell transplantation capacity to restore striatal dopamine transmission and connectivity and MS improvement was investigated [223,224]. Although the first results suggested that it could induce side effects [225], advances in stem cells, which can develop into DA neurons after grafting in animal models, might provide a new opportunity for cell transplantation as a therapy for PD [225,226].

Nonetheless, α-Syn aggregation has become the most investigated target in the search for putative therapies for PD. Different strategies have been explored, including *SNCA* gene-silencing to reduce the neuronal levels of α-Syn, strategies to increase the clearance of aggregated α-Syn by stimulating autophagic or proteasomal activities, and agents that prevent the formation and/or spreading of toxic aggregated structures [56,227]. Antibodies, vaccines, molecular chaperones, and small molecules are some of the most representative agents to target α-Syn aggregation in CNS and PNS. The difficulty for protein-based drugs to cross the BBB and the possibility of developing collateral immunological reactions make small molecules one of the preferred options in PD drug development [228,229].

### 7.1. Polyphenolic Scaffolds

**Figure 6:**
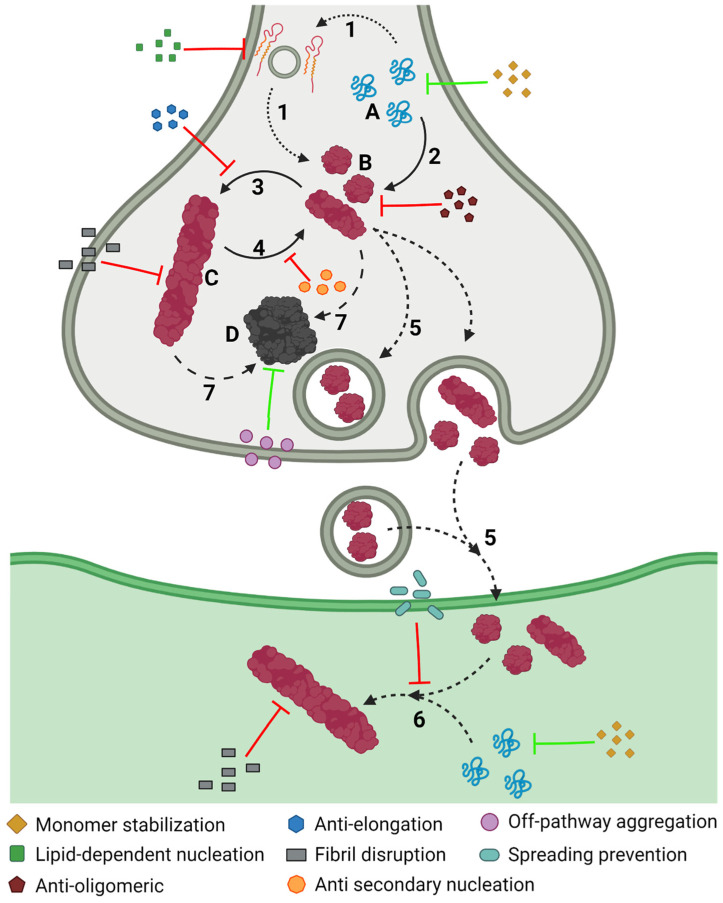
Inhibiting α-Syn aggregation. Schematic representation of the different mechanisms, illustrated by coloured symbols, available to prevent the aggregation of α-Syn, which follows a slow process that comprises different assemblies of the protein: monomeric (**A**), oligomeric (**B**), and fibrillar (**C**), or amorphous (**D**) aggregates. This process comprises different steps during the development of PD: protein–lipid interaction (1), oligomerization (2), fibril elongation (3), secondary nucleation (4), transmission (5), seeding (6), and amorphous aggregation (7).

**Figure 7:**
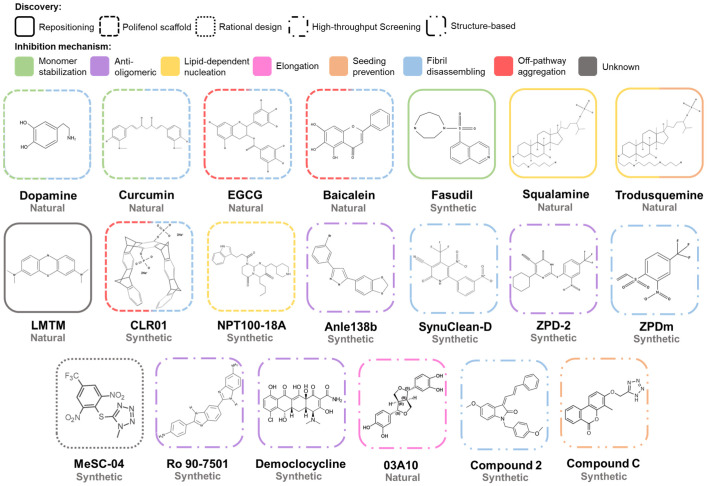
Chemical structures of inhibitors of α-Syn aggregation. Molecular structures of the most relevant modulators grouped by molecular class and mechanism of action. Abbreviations: EGCG, epigallocatechin-3-gallate; LMTM, leuco-methylthioninium bis(hydromethanesulphonate).

The aggregation of α-Syn is a complex process that involves different conformations that small molecules could target to interfere with this process (Figure 6). Accordingly, there are many chemically diverse compounds discovered by different methodologies, which exhibit different mechanisms of action to target α-Syn aggregation (Figure 7). 

The first analyses of the modulators of α-Syn aggregation focused on natural compounds. A pioneering study of 169 molecules revealed that catecholamines, such as dopamine, *L*-DOPA, epinephrine, or norepinephrine [230], interfered with the aggregation process of α-Syn. Particularly, dopamine-oxidized derivates redirect the aggregation of α-Syn to form off-pathway structures [230]. The mechanism behind their inhibitory effect is still unclear; covalent interactions with tyrosine or lysine residues and methionine oxidation of α-Syn by the compounds are some of the multiple proposed mechanisms [231,232,233]. Non-covalent interactions with the _125_YEMPS_129_ sequence at the *C*-terminal region of the protein have also been proposed as the inhibitory basis of catechols [234,235,236,237,238]. Nevertheless, these compounds not only did not prevent the toxicity of α-Syn aggregates, but they stimulated the formation of new toxic species in animal models [239,240].

Polyphenols comprise the largest group of assayed natural compounds, with curcumin, baicalein, myricetin, epigallocatechin-3-gallate (EGCG), ferulic acid, caffeic acid, protocatechuic acid, and gallic acid being the most relevant molecules [241,242,243,244,245,246,247,248]. Polyphenols seem to interact preferentially with the charged and disordered α-Syn *C*-terminal region, inducing the formation of non-toxic off-pathway aggregates [246,248,249], rearranging preformed toxic structures, or dismantling mature fibrils [241]. Ferulic acid was the first polyphenolic structure described as a fibril-disrupting agent [241]. Some compounds exhibit a combination of two or more of these activities [246,250,251,252]. It is worth mentioning that some polyphenols, such as myricetin and curcumin, have been recently described to inhibit α-Syn liquid-to-solid transition in LLPS condensates. Adding these polyphenols to the condensates did not impact their morphology, but they increased the protein solubility, preventing amyloid transition and disentangling the preformed aggregates [253,254]. Structure–Activity Relationship (SAR) analysis of multiple phenolic variants with inhibitory activity revealed that the phenyl group alone does not prevent fibril formation [255]. The inhibitory potential of polyphenolic structures resides in the number (trihydroxybenzoic acid > dihydroxybenzoic acid > monohydroxybenzoic acid), position, and conjugation of the hydroxyl groups at the benzoic acid scaffold [255]. The consecutive arrangement of these polar moieties results in a better inhibitory capacity [255]. Although several of these compounds have been analyzed in animal models of PD showing neuroprotection and the moderation of motor deficits [256,257,258], most of these studies were performed in neurotoxin-induced models of PD [259,260,261]. For example, EGCG has been analyzed in MPTP mice models of PD, in which the compound, as many other polyphenols in similar models [262,263,264,265,266,267,268], exerted a neuroprotective effect [269]. These models do not allow the association of the treatment-mediated improvement to an anti-aggregational effect of the compounds, and the anti-oxidant and anti-inflammatory effects of polyphenols could be responsible for preventing the neurotoxin-induced impairments [258,269]. In this context, curcumin has a special status, as it is one of the few polyphenols tested in transgenic animal models of PD, resulting in motor and behavioral improvements, but without an apparent reduction of protein aggregates [270]. Phenolic molecules are still attracting interest as inhibitors of α-Syn aggregation; ellagic acid has been recently described as a potent modulator of α-Syn unseeded and seeded polymerization and lipid-mediated aggregation in two independent works [271,272]. The compound interacts with monomeric protein, while remodeling and disrupting oligomeric and fibrillar structures, thus precluding the formation of toxic species [271]. However, one of the analyses suggested that cellular protection against α-Syn derived toxicity could be instead related to the restoration of autophagic clearance [272].

### 7.2. Repositioned Compounds

Developing new drugs constitutes a complex process with high attrition rates, substantial costs, and a slow pace. In this context, repositioning or repurposing already approved molecules to develop new treatments for both common and rare diseases has become an attractive strategy. Drug repositioning significantly reduces the risk of failure, the time required for drug development, and the cost of the process. Fasudil is a good example of an attempt to reposition a drug for PD by targeting α-Syn aggregation. This compound is a human Rho kinase inhibitor that has been approved for therapy in cerebral vasospasm and glaucoma [273] with the capacity to cross the BBB and exerts neuroprotection in MPTP-treated mice [274]. This activity was initially considered to derive from inhibiting Rho kinase in the brain [274]. However, further studies demonstrated that fasudil interacts with the aromatic side chains at the *C*-terminal domain of monomeric α-Syn, preventing the nucleation and elongation processes and reducing the intracellular accumulations of α-Syn in a cellular model of PD [275,276]. Remarkably, the administration of fasudil in A53T mice models [277] and AAV-mediated rat models of PD [278] reduced the α-Syn deposits and induced cognitive and behavioral improvements [275,279].

Methylthioninium chloride (MTC), also known as methylene blue, and leuco-methylthioninium bis(hydromethanesulfonate) (LMTM), a reduced stable form of methylthioninium (MT) with a greater absorption rate than MTC has, are other compounds whose repositioning for PD treatment has been studied. Both molecules reported an interesting inhibitory effect in the aggregation of amyloid β (Aβ) and Tau proteins, which are involved in AD [280,281], by disrupting the pre-formed Tau fibril and blocking the Tau–Tau interaction [282]. Studies with these compounds demonstrated a larger inhibitory effect using MTC in vitro, but LMTM presented a greater potential in vivo in terms of dosage and bioavailability. Due to these results, MTC and LMTM have been tested in clinical phases II and III for AD, achieving promising results [283,284,285]. The inhibitory potential of MTC and LMTM observed in Tau encouraged researchers to test whether these compounds could also prevent the aggregation of α-Syn [242]. The incubation of α-Syn with these phenothiazines was translated into a reduction of the in vitro aggregation of α-Syn by increasing its solubility. LMTM treatment in cellular models of PD resulted in a significant decrease in the formation of intracellular aggregates without any effect in terms of protein expression [286]. Moreover, the oral administration of LMTM in mice models of PD [287] demonstrated a reduction of the number of positive α-Syn cells, with a high distribution pattern and without observed side effects, inducing a behavioral improvement. Overall, the LMTM treatment induced a normalization effect on transgenic mice in a dose-dependent manner, correlating with the observed reduction of intra-cellular α-Syn aggregates.

Additionally, squalamine, a steroid-polyamine-conjugated compound [288] first found in *Squalus acanthias* [289] and which presents anti-microbial [290,291] and anti-angiogenic properties [292], has been described as a non-canonical inhibitor of α-Syn aggregation [293]. Squalamine behaves as a cationic lipid that interacts with the inner leaflet of the plasma membrane and destabilizes protein–lipid contact [294,295,296], which affects the initialization of α-Syn aggregation [297]. When α-Syn was incubated in the presence of lipid vesicles and squalamine, the typical α-helical conformation of α-Syn was lost, while the random coil conformation of soluble monomer emerged [293]. Nuclear magnetic resonance (NMR) analysis suggested a weak interaction between α-Syn and squalamine at the *C*-terminal, whereas the interaction between α-Syn and the lipid vesicle took place at the *N*-terminal [293]. However, when squalamine, vesicles, and α-Syn were mixed, NMR revealed a reduction of the interaction α-Syn vesicle, without any evidence of a squalamine–α-Syn binding [293]. According to these results, squalamine seems to reduce the interaction of α-Syn with the vesicles by competing for binding sites on the surface of the lipid structures [293]. As a result, squalamine reduces α-Syn aggregation and oligomeric-mediated toxicity by decreasing the number of oligomers bound to the membranes [293,298]. Moreover, the compound significantly decreased the number of intracellular inclusions, without any effect on α-Syn expression, and improved the motility capacity of a *Caenorhabditis elegans* model of PD [293].

Structural similarities observed between trodusquemine and squalamine suggested that this compound could also prevent α-Syn lipid-mediated aggregation. Trodusquemine is an aminosterol (a polyamine-steroid) with the potential to cross the BBB and stimulate the regeneration of injured tissues in vertebrates by recruiting stem cells, but without affecting the growth of the tissue [299]. As observed with squalamine, trodusquemine prevented the aggregation of α-Syn in the presence of lipid vesicles by displacing α-Syn monomers from the surface of the vesicle where they are bound [300,301]. The CD measurements confirmed a reduced α-helix composition and increased the random coil content [300]. However, the obtained data suggested a more complex inhibitory process than that of squalamine, involving the displacement of α-Syn monomers from the lipid vesicles and the interaction with aggregation intermediates [300]. To further determine whether trodusquemine impacts the elongation or the secondary nucleation, the authors incubated monomeric α-Syn with fibrils at different conditions [176,302,303]. The assays demonstrated that trodusquemine prevented fibril amplification when the reaction is governed by fibril secondary nucleation, but it did not prevent fibril elongation [300]. This inhibitory capacity seems to be related to its binding to the surfaces of amyloid fibrils, displacing monomeric α-Syn from them [300]. In addition, trodusquemine also reduced the toxicity of oligomeric structures due to the displacement of these toxic species [205,304] from the surface of the cellular membrane [301], where they tend to be bound, a phenomenon that correlates with their toxicity [305]. The in vivo analysis in a *C. elegans* model of PD demonstrated that the compound reduced the formation of intracellular inclusions, improved the motility up to similar levels to the healthy controls, and increased longevity, protecting against aggregation-induced toxicity [300].

### 7.3. Compounds Developed by Rational Design

Rational design is another approach to develop pharmacological therapies aimed at specific regions or conformations of a protein. Although the intrinsically disordered nature of α-Syn complicates the exploitation of this strategy, targeting the residues involved in those intermolecular contacts that induce oligomerization, dimerization, or fibrillation has been a successful approach. One of the first rationally designed structures was a molecular tweezer named CLR01, which presents a particular curved structure with a negatively charged cavity. These properties allow non-covalent interactions to occur with Lys10 and Lys12 in the *N*-terminal domain, which translates into a reduction of α-Syn aggregation and the disassembling of preformed fibrils, presumably stimulating the formation of off-pathway oligomers [306]. These studies suggested a link between the number of aromatic scaffolds and the inhibitory potential of a given molecular tweezer, as demonstrated by the absence of the inhibitory potential of CLR03, a shorter variant of CLR01. Further analysis of the inhibitory mechanism of CLR01 demonstrated that the molecular tweezers bound preferentially to the *N*-terminal region of monomeric α-Syn, but also oligomers [307]. This binding alters the hydrophobic and electrostatic interactions that drive protein aggregation and increases the protein’s reconfiguration rate [307]. Cellular studies of the inhibitory capacity of CLR01 in two different models suggested that the molecular tweezer could reduce the toxicity of endogenously expressed and aggregated α-Syn and decrease the administration’s toxic effect of exogenous α-Syn oligomers, increasing the cell viability [306]. In vivo, an analysis using a zebrafish model of PD [308] revealed that CLR01 ameliorates α-Syn-induced damage by reducing the formation of α-Syn neuronal clumps [306]. Furthermore, intracerebroventricular and peripheral administrations of CLR01 in mice models of PD [309] significantly improved the motor behavior of the mice by inducing the formation of off-pathways oligomers and not directly affecting the α-Syn deposits [310]. A more detailed analysis using α-Syn over-expressing mice models for PD [311] and MSA [312] revealed that CLR01 administration increased the neuronal survival rate and improved the motor behavior [313,314]. Interestingly, CLR01 has also shown an inhibitory potential in the aggregation process of different amyloid proteins, such as Aβ40 and Aβ42, Tau, TTR, or PrP, both in vitro [315] and in vivo [316].

Another example of a rationally designed molecular chaperone was developed by the Neuropore and UCB Pharma companies. Based on the assumption that α-Syn dimerization on the membrane surface is crucial for the oligomerization of α-Syn [317,318], they performed a molecular dynamics analysis that revealed a dimerization-responsible pharmacophore region located on residues 96–102 at the *C*-terminal domain. They developed a chemical library with 34 peptidomimetic compounds to target that region and identified NPT100-18A as the most promising candidate [319]. NPT100-18A interaction with the pharmacophore region reduced the number of α-Syn aggregates and increased the monomeric release in vitro by reducing α-Syn interaction with liposomes, thus precluding oligomer formation in the lipid membranes [319]. NPT100-18A reduced the aggregation and its associated toxicity in a primary neuronal cell system overexpressing either WT or E83R α-Syn. Moreover, NPT100-18A oral administration in E47K transgenic mice model exerted a significant neuroprotective effect in multiple brain areas, such as the neocortex or the hippocampus, by reducing the formation of oligomeric species. The analysis of the substantia nigra of these animals revealed a moderated improvement after NPT100-18A administration [319]. Unfortunately, the pharmacokinetic analysis of the compound revealed poor BBB permeability and a very low concentration in the brain [319]. To overcome such a limitation, Neuropore developed NPT200-11, an NPT100-18A derivate with significantly increased BBB permeability. The oral administration of NPT200-11 resulted in brain concentrations of 10 mg/kg, but this increased availability in the brain did not translate into a significant improvement in the symptomatology compared with that of the original molecule [320]. Still, NPT200-11 completed a clinical Phase I trial. Another compound discovered in a cell-based assay by the Neuropore company was NPT520-34 [321]. This molecule presented excellent bioavailability and brain penetrance and increased the levels of LC3, a protein involved in protein clearance, in mice models. Importantly, when it was administered in mice models of PD, NPT520-34 significantly improved PD-related symptoms, reducing PK-resistant α-Syn and normalizing the levels of dopamine transporter, translocator protein, and Toll-like receptor 2. Moreover, the treatment also ameliorated the gait abnormalities associated with NMS of PD [321].

### 7.4. Compounds Derived from High-Throughput Screenings

High-throughput screening (HTS) of large libraries of compounds stands out as a strategy for discovering new active molecules. They require optimized protocols to reduce the cost and time [322]. Regardless of the heterogeneity of protein aggregation, several groups have successfully optimized the α-Syn aggregation protocols and/or integrated new detection systems to identify new chemical chaperones of α-Syn aggregation [323,324,325,326]. Anle138b is an excellent example of an anti-aggregational compound identified with this strategy after two rounds of screening [323]. The first screening step analyzed the ability of ~20,000 chemically diverse drug-like structures to prevent the aggregation of prion proteins (PrP), with 3,5-diphenyl-pyrazole (DPP) being the one with the highest activity level. In a second screening, the authors developed 150 DPP derivates obtained by SAR analysis to retain brain permeability and anti-aggregation activity. In this assay, anle138b emerged as the best candidate against PrP and α-Syn aggregation. This molecule did not present a significant interaction with monomeric α-Syn, but a high binding affinity with a hydrophobic pocket in oligomeric assemblies. The interaction with oligomeric α-Syn precludes the formation of β-sheet interactions, thus avoiding further amyloid aggregation. A recent structural analysis (including CryoEM, solid-state NMR, and solution NMR) has evinced that anle138b binds into the inner cavity of the lipidic fibrillar structure by interacting with Ile188, G68, and G86 [327]. This interaction, driven by polar contacts, causes local structural modifications, altering the structural fluctuations of residues close to the inner cavity [327]. The oral administration of anle138b in three different mice models of PD ameliorated PD-related symptoms such as motor activity, gut motility, neuroprotection, and survival [323,328]. Significantly, the A30P transgenic mice model made it possible to demonstrate that the compound can reduce the number of aggregates in the brain, thus ensuring target engagement [323]. The observed therapeutic effect was also detected in symptomatic late-stage rodents, which opens the possibility that the compound could be effective in the advanced stages of the disease [328]. Moreover, the anle138b inhibitory capacity was also observed in MSA animal models, with a neurodegeneration decay when it was administered at the early stages [329] and motor recovery at the latest phases [330]. Preclinical analyses demonstrated that the compound was innocuous and had excellent pharmacological properties, including significant BBB permeability [331]. 

The optimization of another HTS protocol has allowed researchers to analyze a chemical library from Maybridge HitFinder containing more than 14.000 chemically diverse compounds [332] and to report three modulators of α-Syn aggregation: SynuClean-D (SC-D), ZPD-2, and ZPDm [333,334,335]. The first molecule described, SC-D, significantly reduced the in vitro aggregation of WT, A30P, and H50Q α-Syn variants, while impacting the kinetic constants. SC-D was reported to inhibit α-Syn aggregation at substoichiometric concentrations (7:1 α-Syn:compound molar ratio), indicating that it could target aggregated α-Syn structures. A further analysis, including NMR, suggested that the compound was not a binding monomeric protein, but an aggregated species; indeed, when SC-D was added to mature fibrils, these structures were significantly dismantled, correlating with a bioinformatic analysis that suggested that SC-D could be accommodated into amyloid fibrils, close to the NAC domain. Importantly, SC-D was analyzed in vivo using two different *C. elegans* models of PD, in which the overexpression of human α-Syn induces either the formation of muscular inclusions or the degeneration of DA neurons [336,337,338]. Noteworthy, when they were incubated in the presence of SC-D, the worms presented an impressive reduction of the number of aggregates, improved mobility, and neuroprotection [333]. Moreover, a recent study of SC-D activity proved that the compound exerts a conformation-dependent activity as it presented distinctive inhibitory potential against different α-Syn strains [178]. SC-D works as a pan-inhibitor of spontaneous and seeded α-Syn amyloid formation of different polymorphs, partially dismantling them, and eventually, preventing the formation of cellular inclusions by exogenous seeds [178]. Regarding ZPD-2 and ZPDm, both compounds presented structural similarities, with ZPDm being a minimalistic version and one of the few single aromatic ring molecules that display an anti-aggregational activity [339]. ZPD-2 and ZPDm reportedly decreased the aggregation of WT, A30P, and H50Q even at substoichiometric conditions by targeting aggregated structures rather than monomers. However, they differ in their mechanism of action. While ZPD-2 targets the early stages of the aggregation, ZPDm more efficiently targets and disrupts mature aggregates. Moreover, both compounds were reported to be active against different strains. Despite this alternative mechanistic, ZPDm y ZPD-2 could reduce amyloid formation in *C. elegans* models of PD, in which ZPD-2 also exerted a neuroprotective activity [334,335]. This HTS protocol seems biased to detect compounds targeting aggregated structures rather than monomeric proteins, which represents a clear advantage for its potential therapeutic use since they would only target pathological α-Syn assemblies without interfering with the activity of the soluble and functional protein. Interestingly, SC-D and ZPD-2 have been used as scaffolds to rationally design variants with enhanced activity or pharmacological properties [340,341]. More recently, SC-D and ZPD-2 chemical scaffolds were used to generate a library of 34 different compounds obtained through a similarity-based virtual screening filtered to contain exclusively molecules with good drug-like properties [342]. In vitro studies confirmed the inhibitory capacity of MeSC-04 (an SC-D derivate) against α-Syn aggregation. Computational analysis using two different α-Syn recombinant fibrils (PDB: 2N0A and 6FLT) suggested that MeSC-04 established Van der Waals contacts and hydrogen bonds with the α-Syn region comprising residues from A53 to V74 [342].

The optimization of cellular models for α-Syn aggregation has prompted the development of cell-based HTS protocols [343,344]. One of these new protocols operates by measuring the fluorescence resonance energy transfer (FRET) in an HEK293 cell line that expresses α-Syn fused to EGFP, TagRFP, or both [343]. While this model is intended to analyze small aggregates, the formation of large fibrils can also be induced by adding PFFs to the cells. The analysis indicated that three compounds: Ro 90-7501, Demeclocycline HCl, and Bay K 8644, could remodel the oligomeric structure, and thus, increase the cellular life span. The compounds also exhibited protection against PFFs-induced pathology in primary neurons. An in vitro analysis confirmed the inhibitory potential of the molecules in seeded and unseeded polymerization reactions through the interaction with aggregated protein [343]. Another recently developed cell-based HTS evinced the inhibitory potential of 03A10, a naturally occurring molecule [344]. This cellular HTS is based on protein-fragment complementation assays (PCAs), in which the protein of interest is fused to fragments of a reporter protein whose activity is recovered upon binding. In this case, SH-SY5Y cell lines express α-Syn fused to the *N*-terminus or the *C*-terminus of Gaussia luciferase. Accordingly, luciferase recovers its activity upon the self-assembly of α-Syn. The administration of 03A10 decreased the luciferase activity, but it did not impact cell viability [344]. An in vitro analysis confirmed the reduction of amyloid aggregates, while structural probes indicated an interaction with aggregated α-Syn. Molecular docking suggested that the compound could interact with α-Syn fibrils on residues 50–65. Further PCAs in the PC12 cell line transfected with α-Syn fused to fragments of Venus protein indicated that 03A10 reduced PFFs-mediated intracellular aggregation and increased cell viability [344]. Finally, in MPTP and PFFs mice models of PD, the oral administration of 03A10 improved the behavioral deficits, reduced the number of aggregates and the inflammatory response, and modulated the intestinal disturbances [344].

### 7.5. Structure-Based Strategies for Drug Discovery

The lack of high-resolution three-dimensional structures of oligomeric and fibrillar conformations of α-Syn has precluded the rational design of molecules that target these toxic α-Syn species. Nevertheless, recent advances in structural analyses, including CryoEM or solid-state NMR, have prompted the characterization of in vitro formed and patient-derived α-Syn fibrils [166,185,186]. These fibrillar structures have been recently used to design peptides and small compounds that target α-Syn toxic aggregates [345,346,347,348]. In this way, the structure of an in vitro-generated α-Syn fibril (PDB: 2N0A) and a set of 43 previously described diverse ligands for α-Syn fibrils served to generate a ligand-based pharmacophore modelling and to execute 3D-QSAR studies [346]. Based on the best α-Syn fibrils pharmacophore model (where residues L43, L45, V48, and H50 played a key role), ten indolinone derivates were synthesized and analyzed. The compounds exerted an inhibitory activity in vitro that correlated with their predicted binding and activity. Indeed, the two top-ranked molecules reached levels of inhibition similar to those of EGCG or curcumin [346]. Another structure-based protocol used a two-step computational analysis on top of a recombinant rod-like α-Syn fibrillar structure (PDB: 6CU7) to obtain modulators that target the secondary nucleation process [348]. During the first step, two putative binding pockets were observed in the core and on the surface of the fibril, which involved H50 and E57. As the surface of the fibril acts as the catalytic region for secondary nucleation, this pocket was selected to perform two docking analyses with more than two million CNS penetrant compounds. A docking analysis suggested 1000 candidates that were clustered in 79 groups, and 67 representative molecules were further analyzed. In vitro validation confirmed the inhibitory potential of five of these molecules with a concentration-dependent activity and fibril interaction mediated by the aromatic moieties [348]. The further experimental evaluation confirmed the capacity of the compounds to bind on α-Syn fibril surfaces, rather than the ends specifically, and to prevent and delay oligomeric formation [348]. 

## 8. Conclusions

Protein misfolding and aggregation have been unequivocally linked to neurodegeneration and PD development. This disorder is traditionally characterized by MS, such as rigidity, tremor, bradykinesia, and postural instability. However, it also involves NMS, such as sleep disorders, cognitive impairment, depression, anxiety, pain, or dementia, which significantly impact patients’ quality of life. Both genetic and environmental factors have been found to play a significant role in the onset and progression of PD, with mutations in genes such as *SNCA*, which encodes α-Syn, as one of the paradigmatic examples. Following the discovery of α-Syn as the primary component of fibrillar aggregates in LBs and LNs, it has been widely accepted that targeting the aggregation of this protein could be a promising therapeutic strategy to treat PD.

This review discussed various recent approaches to modulate α-Syn expression and aggregation, including gene therapy and cellular transplantation. A promising area of research is the modulation of aggregation via small molecules. Firstly, catecholamines such as dopamine, *L*-DOPA, epinephrine, or norepinephrine were shown effective in interfering with α-Syn aggregation. In the following years, many other compounds with anti-aggregational properties were discovered. Among them, polyphenols represented the most prominent chemical family of molecules. These compounds interact with α-Syn aggregates and destabilize them by disrupting the hydrogen bond network of the β-sheet, reducing α-Syn self-assembly propensity, and inducing the formation of non-toxic off-pathway aggregates. Interestingly, some polyphenols have been found to inhibit α-Syn LLPS, which has been proposed as a critical step in forming toxic aggregates.

Developing new drugs is often a complex process that is highly expensive and time-consuming. Thus, repositioning the already approved molecules, such as Fasudil, has become an attractive strategy for developing novel molecules targeting α-Syn aggregation. MTC and LMTM are two other related repurposed molecules displaying sound inhibitory effects in vitro against α-Syn aggregation. LMTM showed more significant potential in vivo in terms of dosage and bioavailability. Squalamine and trodusquemine have been repositioned for PD due to their potential to prevent α-Syn lipid-mediated aggregation.

The rational design approach has also been successful in developing pharmacological therapies targeting α-Syn aggregation, such as the molecular tweezer CLR01 or the peptidomimetic compound NPT100-18A. Both CLR01 and NPT100-18A have limitations in terms of their brain bioavailability. NPT200-11 and NPT520-34 were second-generation molecules developed to demonstrate significantly increased BBB permeability. These two molecules have completed clinical Phase I trials and have shown promising results in reducing symptoms and improving neuroprotective effects in animal models of PD.

HTS of large libraries of compounds is an unbiased way to discover new active molecules to inhibit α-Syn aggregation. For example, anle138b, SC-D, ZPD-2, and ZPDm resulted from large computational/in vitro screenings, whereas molecules such as Ro 90-7501, Demeclocycline HCl, Bay K 8644, or 03A10 were identified in more sophisticated, but less processive, in cell screening assays. Irrespective of the methodology employed for their discovery, almost all of these molecules demonstrated effectiveness in animal models of PD.

Structure-based drug discovery strategies involve using a target protein’s known three-dimensional structure to design molecules that specifically bind to and disrupt its function. The CryoEM resolution revolution and methodological advances in solid-state NMR offer an unprecedented opportunity to apply this classical approach in the field of amyloids. Recent studies exploiting atomistic α-Syn fibril targeted approaches have already demonstrated the benefits of structure-based strategies to develop more potent and selective molecules that dock at specific fibril cavities.

Overall, this review illustrates the amazing progress made in understanding and targeting PD from a protein aggregation perspective. Although we still do not have small molecules that have successfully met their primary endpoints in clinical trials, it is also true that they have turned out to be invaluable tools for understanding the molecular mechanisms behind this devastating disease.

## Figures and Tables

**Table 1:** The genetics of PD. Summary of the different gene involved in PD and their role in the development of the pathology.

Gene	Role in PD
*SNCA* (α-Synuclein)	Protein aggregation Prion-like transmission Synaptic function and dopamine transmission
*GBA* (Glucocerebrosidase)	Lysosome mediated autophagy pathway
*LRRK2*	Neurite structure Protein and membrane trafficking Lysosome-mediated autophagy pathway Synaptic function and dopamine transmission
*MAPT* (Tau)	Protein aggregation Neurite structure
*VPS35*	Protein and membrane trafficking Lysosome-mediated autophagy pathway
*DNAJC13* (REM-8)	Protein and membrane trafficking Lysosome-mediated autophagy pathway
*GAK*	Protein and membrane trafficking
*RAB7L1*	Protein and membrane trafficking
*RAB39B*	Protein and membrane trafficking
*Parkin*	Ubiquitin-mediated proteasome Mitochondrial dysfunction and mitophagy
*FBX07*	Ubiquitin-mediated proteasome
*SCA3* (Ataxin-3)	Ubiquitin-mediated proteasome
*PINK1*	Mitochondrial dysfunction and mitophagy
*DJ-1*	Mitochondrial dysfunction and mitophagy
*CHCHD2*	Mitochondrial dysfunction and mitophagy
*POLG1*	Mitochondrial dysfunction and mitophagy
*SREVF1*	Mitochondrial dysfunction and mitophagy
*ATP12A2*	Lysosome-mediated autophagy pathway
*SCARB2* (LIMP-2)	Lysosome-mediated autophagy pathway
*SYNJ1* (Synaptojanin 1)	Synaptic function and dopamine transmission
*GCH1*	Synaptic function and dopamine transmission
*STX1B* (Syntaxin-1B)	Synaptic function and dopamine transmission

## Data Availability

Data sharing is not applicable to this article.
